# On the Phylogenesis of Executive Functions and Their Connection with Language Evolution

**DOI:** 10.3389/fpsyg.2016.01426

**Published:** 2016-09-21

**Authors:** Ines Adornetti

**Affiliations:** Department of Philosophy, Communication and Performing Arts, Roma Tre UniversityRome, Italy

**Keywords:** executive functions, discourse processing, global coherence, language origin, narrative, prefrontal cortex, stone toolmaking, traumatic brain injury

## Introduction

T term Executive Functions (EFs; also executive control or cognitive control) refers to the higher order cognitive processes involved in the regulation of goal-oriented actions in complex contexts and non-routine situations (Gilbert and Burgess, 2008). EFs are crucial for the temporal organization of purposive behaviors, language and reasoning (Fuster, 2008). There is no single taxonomy of EFs that scholars generally agree upon (Jurado and Rosselli, 2007). The various models proposed in the literature suggest that EFs capacities entail several key components, including flexibility, planning, monitoring, working memory, and inhibition (Ardila, 2008). Although a dynamic and flexible network involving several cortical and subcortical brain regions mediates EFs, there is a consensus that their main neural substrate is the prefrontal cortex (PFC) (Fuster, 2008). Specifically, the dorsolateral region of PFC is generally involved in classic EFs such as planning, problem-solving and some working memory operations, whereas the orbitofrontal region is more closely associated with the regulation of emotions and social behavior.

Neuroscientific (Barkley, 2001) and comparative (Hills, 2011; Völter and Call, 2014) studies have dealt with the issue of the evolution of EFs. The evolution of EFs for the later PFC regions and their connections with language origins has been the subject of particular interest (Risberg, 2006). Some studies have suggested that the emergence of EFs occurred recently in the evolution of *Homo sapiens* (Ardila, 2008; Coolidge and Wynn, 2009). According to Coolidge and Wynn (2009) and Wynn and Coolidge (2007), modern humans evolved an enhanced working memory capacity that fostered EFs 32,000 years ago, enabling complex contingency planning, abstract reasoning and innovation. Ardila (2008) has proposed that EFs appeared 150,000 years ago in connection with the advent of language grammar. According to Ardila (2016), the temporal organization of behavior (the core function of EFs) comes from the perception of actions that is correlated with the grammatical ability to use verbs and represent these actions:

Temporality means “before” and “after,” that is, something that changes, or develops or moves, that is, an action. (…) the “perception of actions” would represent a single preadaptation both for grammatical language and for meta-cognitive executive functions (Ardila, 2016, p. 2).

In this paper, I advocate a different scenario for the phylogenesis of EFs and their connection with language evolution. By reviewing studies coming from Evolutionary Cognitive Archaeology (ECA), I suggest that EFs evolved in the context of toolmaking before the appearance of *Homo sapiens*. By virtue of this, I treat the issue of the relationship between EFs and language evolution in a different manner to the models that assigned a prominent role to grammar. I hypothesize that language could have had a narrative origin and that the study of the evolution of EFs together with the investigation of the role of EFs in language processing can corroborate this hypothesis.

## Executive functions and stone toolmaking

ECA analyzes the archeological record to determine the course, the timing and the factors driving the evolution of the mind (Wynn, 2009). Though its epistemological character is controversial since “the concept of empirical testability introduced by Bell (1994) is insufficient” (Garofoli and Haidle, 2014, p. 9), it has been proposed that ECA can be founded on a new method of testability: a deductive mapping from networks of theories (Garofoli and Haidle, 2014). Theories on lithic industries represent a piece of this general puzzle. They are relevant to my proposal since they underline the crucial role of hierarchical structures in toolmaking (e.g., Pelegrin, 2005).

According to Mahaney (2014), two broad approaches characterize the study of lithic technology within ECA: those that analyze knowledge and those that investigate the processing systems that make that knowledge possible. Concerning the former, the *chaîne opératoire* approach (Leroi-Gourhan, 1993) is very important; it examines knowledge by describing the action sequences needed to make a particular artifact, highlighting the hierarchical organization of knapping in terms of goals and sub-goals. Hierarchical structure is cognitively relevant since it implies superordinate representations abstracted from, and maintained over, the course of multiple subordinate events (Stout, 2011). As such, it implicates processes associated with the distinctive response properties and anatomical connections of PFC (Badre and D'Esposito, 2009).

Regarding the processing systems, Mahaney (2014) suggested that Wynn (1993) three-layer model of tool behavior provides a framework to investigate the neurocognitive processes underlying technical knowledge. The layers are biomechanical (affordances of the anatomy of the stone knapper), sequence construction (process of concatenating actions to achieve a goal), and problem-solving/cognitive control (processes that guide and select sequentially structured actions). Work by Gowlett (2006), Moore (2010), and Uomini and Meyer (2013) are within this framework. In particular, according to Mahaney (2014, p. 183), Wynn's model is supported by neuroimaging studies of modern knappers contrasting Oldowan and Acheulean replication (Stout et al., 2008; Stout, 2010). Chronologically, Oldowan and Acheulean are the second and the third known lithic industries in the archeological record. The earliest stone artifacts, dated to 3.3 million years ago (mya), have been recently discovered at Lomekwi (Harmand et al., 2015). Oldowan flaking (from 2.6 to 1.4 mya) (Semaw et al., 1997) is characterized by the production of sharp stone flakes obtained by striking one stone with another. Acheulean toolmaking started 1.7 million years ago with *Homo ergaster* and *Homo erectus* (Lepre et al., 2011). The early Acheulean tool is a bifacial irregular reduction similar to developed Oldowan tools. The Late Acheulean (~ 0.7 to 0.25 mya) hand axe is a refined bifacial shaping made by modeling a large stone on both sides until an almond-shaped symmetrical stone is obtained. Neuroimaging studies involving modern humans (Stout et al., 2008, 2015) suggested that the evolution of toolmaking during human phylogeny can be tied to the enhancement of EFs and that a crucial advance in this regard occurred with the appearance of Late Acheulean industry. The steps required to build Late Acheulean hand axes imply the contribution of the lateral PFC (Stout et al., 2008, 2015), an area crucial for the hierarchical representations of sequential actions (Badre, 2008). The conclusion that can be drawn from these studies is that EFs evolved in the context of toolmaking in some of the early species of genus *Homo.*

## Executive functions and language evolution

The main result obtained by positioning the evolution of EFs at the beginning of genus *Homo* is the possibility to consider EFs as autonomous and independent from language. Such positioning allows the development of a language model in which EFs are a precondition for the origin of human communication, rather than an outcome of grammar. To construct such a model, two steps are required: (1) the analysis of the involvement of EFs in language functioning; and (2) the construction of a language evolution model in line with the role that EFs plays in language processing. Regarding the first step, I show that EFs play a key role in the pragmatic processing of narrative; regarding the second, I propose a proto-narrative account of language phylogenesis.

Numerous studies have substantiated the role of EFs in language processing (Bialystok et al., 2004; Weiss et al., 2010). Relevant to my proposal of a narrative origin of language are investigations that have shown the crucial involvement of EFs in discourse processing both in typical brain subjects (Cannizzaro et al., 2016) and in neuropsychological populations (Sirigu et al., 1998). Among these, studies on the discursive abilities of subjects with traumatic brain injury (TBI) resulting from damages to the PFC, specifically to the lateral regions (the same involved in Late Acheulean toolmaking), attest that TBI patients have deficits in action planning and organization (Zalla et al., 2001), as well as severe communication impairments (McDonald et al., 1999). Concerning the former, they are not able to organize and execute appropriate plans of actions; concerning the latter, they display a dissociation between the abilities underlying the level of macroanalysis (pragmatic and discourse processing between sentences) and those underlying the level of microanalysis (lexical and syntactic processing within a single sentence) (Glosser and Deser, 1990). TBI patients have impairments in managing global coherence—a specific property of discourse that concerns the manner in which a narrative is organized with respect to an overall goal, plan, theme, or topic. In spite of being able to process the lexical and syntactic aspects of individual sentences, TBI patients have problems in coherently connecting sentences during the flow of discourse (Marini et al., 2011, 2014; Coelho et al., 2012). As a result, their narratives appear confused and disorganized. The discursive deficits of TBI patients have been tied to EFs impairments (Mozeiko et al., 2011; Marini et al., 2014). Such a finding is highly relevant to my argument, as it suggests that the processing of discourse coherence does not depend on linguistic (i.e., grammatical) abilities, but relies instead on the correct functioning of EFs (Adornetti, 2014a).

It has been proposed that language disorders provide hints about the evolution of the neural substrates of linguistic abilities (Code, 2011; Ardila, 2015). Therefore, studies on the discursive deficits of TBI subjects together with neuroarcheological investigations might be used to clarify the cognitive prerequisites for linguistic behavior, allowing us to connect the involvement of EFs in language processing with the issue of its origins. A starting point is the adhesion to a narrative model of language origin according to which the capacity to produce discourse precedes the capacity to produce phrases or words (cf. Ferretti, 2016). In this scenario, I hypothesize that the executive processes that underpinned toolmaking also allowed the emergence of discourse. Indeed, knapping and narrative production have some similarities: they are both organized in a top-down hierarchical fashion with the overall goals guiding the organization of the sub-goal sequences (see also Mahaney, 2014, p. 175). Indeed, toolmaking requires that the knapper monitor how past, current and future actions relate to one another as a means to achieve the final goal. In storytelling, similarly, it is necessary to describe a sequence of connected events (maintaining information across the sentences and relating them to each other in a sequential manner) in order to reach the goal of the story (i.e., its conclusion that is necessary to grasp the narrative gist) (Ferretti et al., 2013). From this perspective, it is possible to conceive of discourse production and toolmaking activity as specific cases of the more general execution of goal-oriented behaviors. Consequently, the activation of lateral PFC in knapping replication experiments discussed earlier could be analogous with the role that the region plays in the discursive level of language.

Pursuant to these considerations, I suggest that an important event in the evolution of language was the exaptation of the network of EFs for communicative purposes. This exaptation provided the foundation for the rise and gradual evolution in the hominin lineage of a communicative system based on a kind of proto-discourse—connected sequences of communicative actions using verbal and other resources- governed by coherence (Adornetti, 2014b, 2015). The evolution of proto-narrative might have been propelled by the need to produce stories. Although human language shares several features with animal communication (Hauser et al., 2002; Tomasello, 2008; Zuberbühler, 2015), it is plausible to maintain that language contains traits that distinguish it from any other form of communication. Undoubtedly, the ability to tell stories is uniquely human (Thompson, 2010). Indeed, several authors have suggested that storytelling was the initial condition of departure for the evolution of linguistic behavior (Ferretti, 2014, 2016; McBride, 2014; Corballis, 2015). The point to stress here is that narrative does not require a complex code (i.e., grammar) to operate (narrative does not require language at all: Boyd, 2009, p. 159). In view of this, the discourse level (governed by a pragmatic property, such as coherence) precedes the grammar of sentences and words. That said, linguistic grammar is an essential component of human language, and it may be argued that grammar has made storytelling more accurate and efficient. Otherwise stated, it is possible to speculate that the need to share the information included in stories in a more precise way brought grammatical structure to the communicative system. In this sense, pragmatics—in the form of coherent proto-discourses underpinned by EFs—both precedes grammar and is the precondition for its emergence.

## Author contributions

The author confirms being the sole contributor of this work and approved it for publication.

### Conflict of interest statement

The author declares that the research was conducted in the absence of any commercial or financial relationships that could be construed as a potential conflict of interest.
